# Assessing Health-Related Quality of Life in Children With Spina Bifida in Lithuania

**DOI:** 10.7759/cureus.63742

**Published:** 2024-07-03

**Authors:** Faris Ali, Indrė Bakanienė, Hytham Dafalla, Audronė Prasauskienė

**Affiliations:** 1 Trauma and Orthopedics, Lithuanian University of Health Sciences, Kaunas, LTU; 2 Pediatric Rehabilitation, Lithuanian University of Health Sciences, Kaunas, LTU

**Keywords:** quality of life, health-related quality of life, multiple-choice questions, chiari ii malformation, cerebrospinal fluid (csf), central nervous system, spina bifida

## Abstract

Introduction

In recent years, more emphasis has been placed on improving the health-related quality of life (HRQOL) in children with spina bifida (SB). Chronic disability is understood to impact various aspects of the person's life, family, and social functioning, in addition to the specific needs of the disease. The HRQOL is done to assess the patient's quality of life (QOL) in various domains including physical and mental. Back in the 1900s, few children survived SB, whereas today, they almost have normal life expectancy. By understanding the contributing factors to the quality of life (QOL), more targeted interventions can be put in place in order to maximize the psychological and social well-being of these patients.

Aim

The aim of this study is to estimate the health-related quality of life (HRQOL) in Lithuanian children with spina bifida (SB) in relation to comorbidities, level of lesions, and mobility.

Objectives

The objectives of this study are to investigate the HRQOL of Lithuanian children with SB born between 1999 and 2012; to analyze the relation between the HRQOL and its comorbidities, including hydrocephalus, Chiari II malformation, incontinence, and epilepsy; and to determine the relationship of health variables, the level of lesions, and mobility to the HRQOL.

Methods

This was a quantitative cross-sectional study on children with spina bifida across Lithuania to assess the HRQOL. Subjects were chosen and interviewed from various cities including Kaunas, Vilnius, Marijampolė, Gargždai, Biržai, Panevėžys, Palanga, and Alytus. A questionnaire was used as an instrument to measure the HRQOL. The level of lesions, comorbidities, and other health variables were obtained from the medical files and directly from the patient's history.

Results

Regarding the HRQOL, our study population showed the highest scores in the emotional, medical, intellectual, and social domains. The lowest sub-scores were in recreational, vocational, environmental, and then physical domains. We also found that certain comorbidities including hydrocephalus, epilepsy, and incontinence negatively affected the QOL. In our study group, we also found that the ambulatory group scored significantly higher in the overall QOL. However, when comparing the level of lesions to the HRQOL, we found no statistically significant difference.

Conclusion

Positive results were obtained regarding the medical, emotional, intellectual, and social aspects of patients with SB in Lithuania as they scored high in this domain. However, the environmental and vocational domains scored low, suggesting that further examination needs to be carried in these domains. We concluded that having various comorbidities including hydrocephalus and incontinence has negative impacts on the QOL. Patients who suffered from epilepsy had a statistically significant lower QOL. No significant difference was found in the association between the level of lesion and the QOL in our study.

## Introduction

In recent years, more emphasis has been placed on improving the health-related quality of life (HRQOL) in children with spina bifida (SB). Chronic disability is understood to impact various aspects of the person's life, family, and social functioning, in addition to the specific needs of the disease. The HRQOL is done to assess the patient's quality of life (QOL) in various domains including physical and mental. Back in the 1900s, few children survived SB, whereas today, they almost have normal life expectancy [1]. By understanding the contributing factors to the quality of life (QOL), more targeted interventions can be put in place in order to maximize the psychological and social well-being of these patients.

In Lithuania, no study has been conducted on the HRQOL of children with SB. Despite SB being the second most common congenital disorder after Down syndrome by prevalence in Lithuania [2], very few people know about the disease and its long-term effects; SB is caused by both genetic and external harmful factors, which disrupts the normal development of spinal development. Symptoms will vary depending on the level of lesion [2].

In the literature, there are only a few studies performed regarding the long-term prognosis of children with SB. In general, survival and the degree of neurological impairment depend on the level of the spinal segment involved, the severity of the lesion, and the extent of associated abnormalities. Long-term survival into adulthood and advanced age is now common with aggressive treatment and an interdisciplinary clinical approach. The National Birth Defects Prevention Network (NBDPN) and other multistates found that the mortality rates declined with folic acid fortification from the US grain supply in the 1990s [3]. Furthermore, survival at one year of age increased significantly from 20% to 80% following neurosurgical intervention, despite limited knowledge regarding long-term effects [4]. The following main systems are involved in causing mortality in children with SB: renal, cardiorespiratory, and CNS infection; hydrocephalus; and thrombocytopenic purpura [4]. Many of these could possibly be avoided if a timely and adequate treatment is provided.

The majority of the affected children require complex and long-term multidisciplinary care input. Recently, in 2016, a conference was organized in Norway, with the aim of bringing both the Lithuanian and Norwegian spina bifida and hydrocephalus (SBH) associations together to form a partnership [2]. This was successfully done with the help of the nongovernmental organization (NGO) and the European Economic Area (EEA) grant funding. Centers have made special training courses available for Lithuanian nurses and social workers, which will in turn help to improve the training of Lithuanian families with SB [2]. The Scandinavian countries are considered to have one of the best healthcare systems in the world, and hopefully, this new partnership will improve our understanding of SB in Lithuania and encourage more local studies to be carried out.

Various types of questionnaires were used to assess the HRQOL in children with SB compared to healthy children. Examining the literature, the questionnaires that have been used in these studies include the following: the Pediatric Quality of Life Inventory (PedsQL), Schedule for the Evaluation of Individual Quality of Life-Direct Weight (SEIQoL-DW), and Health Utilities Index Mark 3 (HUI3) [5-9], and Parkin et al. (1997) [10]. The SEIQol-DW survey varied greatly from the others in that it used open questions and therefore translates more easily across cultures [6-9].

## Materials and methods

For our study, patients with SB born between 1999 and 2012 were chosen from different cities throughout Lithuania. They were identified from neurology outpatient and ambulatory clinics and from various hospital birth registries. This study included patients with all forms of SB. Seventy subjects that met the criteria were identified; however, only 31 participated in the study. The eligible subjects were invited via invitation letters and follow-up telephone calls with detailed information regarding the study. To reduce the percentage of dropouts due to financial reasons such as travel expenses, an option of home visitations if preferred was offered to parents in the invitation letter sent, especially to those who lived far in rural areas. The Lithuanian University of Health Sciences (LSMU) Bioethics Center issued approval BEC-MF-453 (date of issue: 2016-04-28)

Two different questionnaires were given, which were specific for each age group. The age groups included one for 5-12 years of age and one for 13-20 years of age. In some cases however, due to the age of the child or cognitive impairment, answers were received with the assistance of the parents.

A questionnaire developed by Parkin et al. (1997) [10] was used in the assessment of the HRQOL. It was used to assess the social, emotional, intellectual, financial, medical, environmental, physical, recreational, and vocational domains. Before the subjects received the questionnaire, it needed to be translated from English into Lithuanian. Questionnaires with 44 items were given to the children 5-12 years of age, and questionnaires with 47 items were given to the children 13-20 years of age. The instructions were given for the items to be filled out fully and to "indicate how important each of these statements is to you when considering your quality of life." Each item had to be evaluated on a five-point Likert scale according to importance. The scale ranged from 1 point for "does not apply" to 5 points for "extremely important."

Sensibility

The questions used in this scale were short, simple, and closed-ended. The child answered the questions in a direct manner. A few children that were of the youngest age or had intellectual challenges required assistance from their parents, and these parents were told to answer the questions "from the child's point of view." When the questionnaire was being translated into Lithuanian, some words and phrases were reconstructed to cause less confusion when the questions were answered and evaluated.

Questionnaire scoring

All the questions were added according to what score was chosen from the five-point Likert scale. Differentiation was not necessary, and each question was given equal weight. However, as a consequence of the inconsistency in the number of questions, the results were subscaled to values between zero and 100, where 100 corresponds to perfect QOL, in order to allow for easy and accurate comparability of the statistical analysis.

Statistical analysis

The results of the survey were examined, and various statistical tests were performed using the SPSS version 23 software package (IBM SPSS Statistics, Armonk, NY). T-test was used to compare the mean in two groups and ANOVA to compare the mean scores in three or more groups along with a Bonferroni post hoc test. A P value of less than 0.05 was used as a statistically significant result.

## Results

Population characteristics

A total of 31 children with SB were interviewed between July 2016 and February 2017. Twenty (65%) of these were aged between five and 12 years, and 11 children (35%) were aged between 13 and 20 years. As can be seen in Table 1, there were 18 males (58%) and 13 females (42%) included, the majority of which lived with their parents. The level of lesions was divided into four groups: upper (thoracic lesions), lumbar L1-2 and L3-5, and sacral lesions with a good spread between all levels. Using the "Hoffer ambulation scale," 16 children (52%) were nonambulatory, and 15 (48%) were ambulatory. Of the ambulatory patients, eight were able to walk normally, and the remaining seven had limited mobility, either walking with aids or only part of physiotherapy-guided exercises.

**Table 1:** Patient Characteristics (N=31)

	N (%)
Gender	
1. Male	18 (58%)
2. Female	13 (42%)
Lives with	
1. Father/one parent	27 (87%)
2. Guardians	1 (3%)
3. Care facility	3 (10%)
Level of lesion	
1. Thoracic	8 (26%)
2. L1-L2	7 (23%)
3. L3-L5	10 (32%)
4. Sacral	6 (19%)
Walking (Hoffer scale)	
1. Do not walk	16 (52%)
2. Walk only during physiotherapy	2 (6%)
3. Walk at home with aid	1 (3%)
4. Walk in all environments with aid	4 (13%)
5. Walk normally	8 (26%)

Surgically, 18 out of 31 children (58%) have been treated for hydrocephalus while only four children (13%) for Chiari II malformation. Five children (16%) suffered from epilepsy. Eighteen children (58%) had spinal deformities, four of which were treated surgically. Eighteen children (58%) had hip deformities, three of which were treated surgically. The most common deformity was foot deformities with 21 children (68%) affected, 11 of which had surgical treatment, as shown in Table 2.

**Table 2:** Patient Comorbidities (N=31) Tx: treatment

	N (%)
Hydrocephalus Tx surgically	
1. None	13 (42%)
2. one or more times	18 (58%)
Chiari II malformation Tx surgically	
1. None	27 (87%)
2. Yes	4 (13%)
Bladder catheterization Tx	
1. None	15 (48%)
2. Yes (three or more times)	16 (52%)
Urinary incontinence	
1. None	5 (16%)
2. Sometimes (≤1/week)	5 (16%)
3. Always	21 (68%)
Urology operation	
1. None	30 (97%)
2. Yes	1 (3%)
Syringomyelia Tx surgically	
1. None	30 (97%)
2. Yes	1 (3%)
Epilepsy drug therapy	
1. None	26 (84%)
2. Yes	5 (16%)
Inserting catheter	
1. Alone	11 (35%)
2. With help	23 (65%)
Constipation	
1. None	15 (48%)
2. Tx with medication	6 (20%)
3. Washouts	10 (32%)
Spinal deformities	
1. None	13 (42%)
2. Tx conservatively	14 (45%)
3. Tx surgically	4 (13%)
Hip joint deformities	
1. None	13 (42%)
2. Tx conservatively	14 (45%)
3. Tx surgically	4 (13%)
Foot deformities	
1. None	10 (32%)
2. Tx conservatively	10 (32%)
3. Tx surgically	11 (36%)

Regarding urinary incontinence, 21 children (68%) were affected, and 16 children (52%) used urinary catheters. One child had a surgical bladder reconstruction. Fifteen children (48%) had fecal incontinence, while 16 children (52%) suffered from constipation, of which six were conservatively treated with medication and 10 with a routine washout.

Based on the questionnaire, cognitive deficiency levels were divided into five groups: none, mild, moderate, severe, and very severe. Sixteen children had no intellectual disability, one child had a mild intellectual disability, one had a moderate intellectual disability, and five had severe levels, while in eight children, it was not defined.

None of the children in the study had any of the following comorbidities: chronic pyelonephritis, renal failure, or tethered spinal cord injuries.

HRQOL questionnaire

**Figure 1:**
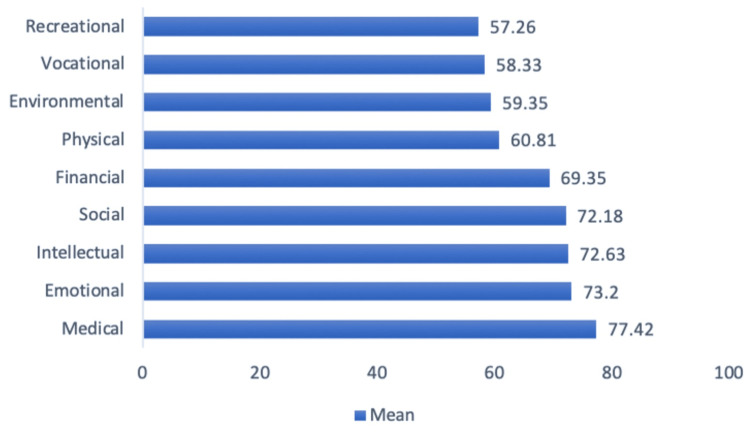
QOL in the Different Domains (Rescaled 0-100) QOL: quality of life

When examining the results of the questionnaire and the various domains assessing the quality of life, it was found that the overall highest scores were in the emotional, medical, intellectual, financial, and social domains, whereas the lowest sub-scores were in the recreational, vocational, environmental, and then physical domains, as shown in Figure 1.

The emotional domain in both age groups scored relatively high. Children in this domain showed that they were able to express their emotions and feel comfortable about themselves. This was seen with patients scoring the highest in being able to "express his/her emotions" and "feel positive about themselves."

The medical domain scored highly in both age groups, and there was an overall high satisfaction regarding the care and respect they receive in the hospital, as well as the level of understanding shown regarding their condition. The patients involved in the study scored high in the intellectual domain with an average of 79.58 in the 5-12 age group, as shown in Table 3. They were satisfied with their school program, with all patients scoring full marks with "receives praises for things that he/she is able to do."

**Table 3:** Comparison of HRQOL in the Different Age Groups (Rescaled 0-100) HRQOL: health-related quality of life

	5-12 age group				13-20 age group	
	Mean	Median	Standard deviation	Standard error of mean	Mean	Median
Social_re	78.12	87.50	25.37	5.67	61.36	62.50
Intellectual_re	79.58	91.67	22.21	4.97	60.00	70.00
Medical_re	81.67	83.33	15.73	3.52	69.70	66.67
Recreational_re	72.75	75.00	21.67	4.85	29.09	20.00
Vocational_re	67.71	79.17	31.21	6.98	41.29	45.83
Financial_re	66.25	75.00	37.41	8.37	75.00	75.00
Environmental_re	63.25	65.00	27.11	6.06	52.27	58.33
Physical_re	66.25	58.33	27.64	6.18	50.91	65.00
Emotional_re	76.50	75.00	15.76	3.52	67.21	67.86
HRQOL_re	73.27	79.55	19.46	4.35	56.04	55.32

In the social domain, the overall average score was 78. The children scored the highest in "support from the family" and scored lowest in relation to having a "special friend" or "someone to confide to outside of their immediate family."

The recreational domain scored relatively high in the 5-12 age group, whereas it scored very low in the 13-20 age group (mean of 73 versus 29, respectively), as shown in Table 3 and Table 4. This could perhaps be explained by the fact that only 25% of children aged 5-12 were wheelchair-bound, compared to 67% in the age group 13-20. In the older age group, they did not participate or feel challenged or encouraged through outdoor or indoor sports activities.

**Table 4:** Comparison of HRQOL in the Different Age Groups (Rescaled 0-100) HRQOL: health-related quality of life

	13-20 age group		Total		
	Standard deviation	Standard error of mean	Mean	Median	Standard deviation
Social_re	19.28	5.81	72.18	75.00	24.45
Intellectual_re	25.59	7.72	72.63	75.00	24.93
Medical_re	12.23	3.69	77.42	79.77	15.51
Recreational_re	23.00	6.94	57.26	65.00	30.41
Vocational_re	31.32	9.44	58.33	54.17	33.30
Financial_re	20.92	6.31	69.35	75.00	32.41
Environmental_re	21.44	6.46	59.35	60.00	25.44
Physical_re	39.23	11.83	60.81	58.33	32.44
Emotional_re	16.27	4.91	73.20	75.00	16.31
HRQOL_re	18.11	5.46	67.16	68.62	20.48

In addition, the environmental domain scored relatively low in both age groups. Children of all age groups did not feel that the outdoor resources were sufficient for their disabilities, scoring the lowest in "the ability to use and access public washrooms."

In addition, the vocational domain also scored relatively low in both age groups. However, children aged 5-12 felt that they "had the chance to study in things he/she is interested in," scoring the highest in that question, whereas children aged 13-20 had less hope, scoring relatively low in "being able to get an education for a job that interests you."

In relation to the different age groups (5-12 years versus 13-20 years), the younger age group scored statistically significantly higher in the overall HRQOL (p=0.022), with the following domains showing statistical difference: intellectual (p=0.034), medical (p=0.037), recreational (p=0.00), and vocational (p=0.032).

There was no difference in the HRQOL between different genders, with only the emotional domain showing statistical difference (p=0.030), with males scoring lower in this domain.

Level of lesion and comorbidities and the HRQOL

Using the ANOVA one-way test for statistical analysis, we found some statistical differences between the level of lesion and the HRQOL in the following domains: vocational, recreational, environmental, physical, and emotional. However, following the application of the Bonferroni correction, there was no statistically significant difference between the level of the lesion and the HRQOL.

In those with Chiari II malformation, there was no statistical difference in the HRQOL. However, there was significant difference found in those with hydrocephalus (p=0.002), with only the social domain showing no difference between the groups.

In relation to mobility and the QOL, those unable to walk scored lower (p=0.002). They scored significantly lower in the physical, recreational, environmental, medical, and vocational domains. Regarding deformity, spinal and hip deformities had a statistically significant difference in the QOL (p=0.000 and p=0.006, respectively), but there was no statistical difference in the overall QOL in those with knee or foot deformity. Spinal deformity affected all domains except for financial in the children's perspective, while hip deformity mainly affected the physical, environmental, recreational, and vocational domains. Foot deformity showed a statistical difference in the emotional, medical, and social domains.

Patients who suffered from epilepsy had a statistically significant lower QOL (p=0.004), with all domains affected except for intellectual, recreational, and environmental.

The children who were affected by urinary incontinence (68%) had statistically significant lower QOL (p=0.000), with lower sub-scores in all domains except for financial. Nonetheless, requiring urinary catheterization did not appear to influence the QOL. Fecal incontinence also had negative effects on the QOL but to a lower extent than urinary incontinence (p=0.009) with only five out of the nine domains affected. With incontinence, the highest significant difference was in the emotional domain.

Furthermore, those who had no intellectual disability scored significantly higher in all domains (p=0.00). Children with upper lesion "thoracic" had a lower QOL in the physical and recreational domain compared to lower level of lesions. Higher lesions also resulted in lower scores for the vocational domain. In the recreational domain, over 80% of the children with sacral and L3-L5 lesions, aged between five and 12 years, scored 5 and had no difficulties in participating in outdoor activities and going out on dates. This was seen in the vocational domain where they scored highly regarding "having the chance to continue to study the things he/she is interested in." In the 13-20 age group, similar findings were seen regarding the physical, recreational, and vocational domains. However, interestingly, it was found that in the children aged 13-20, those with lower lesions scored lower than those with higher lesions in the emotional domain.

## Discussion

Our study is one of the first to look at the HRQOL in children with SB in Lithuania. It is community-based and focused on specific age groups, which can have both pros and cons.

Although the questionnaire we used (Parkin et al., 1997 [10]) is a disease-specific questionnaire that was developed for patients with SB and is more relevant than other questionnaires, it also, however, has some limitations especially when used to assess the physical domain in children that are not mobile and translating the questionnaire from English to Lithuanian, which invalidates the questionnaire in our study. Several questions are not appropriate to measure the physical function of individuals who use a wheelchair, which may have led to biased lower scores for those reliant upon wheelchairs for mobility. Furthermore, the environment also had some influence on certain questions. For example, problems with showering or bathing may be a combination of financial and physical capability rather than just a physical issue alone. Unfortunately, it was not possible to differentiate influences from the environment on the results. However, the questionnaire had good measurement properties and was a good tool for discriminative measures.

Regarding the level of lesions, there was a good but not equal representation of patients with various levels of lesions. We found no statistical difference between the level of the lesions, which is not consistent with some of the other studies. This may be due to the power of the study. One study showed that the QOL scores decreased in the L2 and above lesions compared with L3-L5 [11]. One main limitation of this study is the fact that only the parents were interviewed, and therefore, the QOL scores are from the parents' own perceptions, and this could have significantly affected the results. Another study, "Health outcomes among youths and adults with spina bifida," also showed significant differences, with the lower level of lesions having a better QOL. This study used both HUI3 and Assessment of Quality of Life (AQoL) questionnaires to assess the QOL, allowing it to have a better mean score compared to our study [12].

Our study also suggested that comorbidities including both urinary and fecal incontinence are indeed associated with a decreased QOL. This was the same case in other studies, although different questionnaires were used [6,13,14]. However one study, "Quality of life and continence in patients with spina bifida," did not show any significance between urinary incontinence and the QOL [15]. This might be due to the population of the study with 65% being 18 years of age and older. However, significant improvement to the QOL was shown in their study for those with surgical interventions for continent diversion. Our study was not able to show this difference due to the small sample size as we only had one child with surgical diversions compared to the large sample size of the other study (460 patients).

Also, other comorbidities such as epilepsy decreased the QOL, which reiterates how being affected by multiple conditions can be very challenging to the individual and negatively attenuates the QOL. Unlike other studies, we found that having Chiari II malformation did not make any statistical difference to the QOL, while hydrocephalus did [16]. A potential bias could have resulted from the sample size as only four patients (13%) had Chiari II malformation, compared to 18 children (58%) that had hydrocephalus and surgery.

Regarding ambulation, the ambulatory group scored significantly higher in the overall QOL, which was expected. There was a statistically significant difference in the physical domain, despite the fact that the questionnaire as mentioned was not sensitive to assess the physical domain in patients that are wheelchair-bound, and therefore, the difference might actually still be underestimated. Furthermore, because of the small numbers in our study, we did not differentiate between those that are mobile with aid in our analysis. However, one study, "Self-reported health-related quality of life in children and adolescents with myelomeningocele," showed no significant difference between the QOL and the ambulatory group [17]. Their population sample was relatively small for wheelchair-bound users, with only seven participants compared to 38 subjects being able to walk, which could have had a significant impact on the results [17].

In relation to the physical and vocational domains, they received some of the lowest scores compared to the other domains, and this is consistently seen with other studies [18]. Although some other studies showed an influence between resource availability and the QOL, our sub-scores the for "financial" domain were good, but we had poor scores for the "environmental" domain, where it ranked the second lowest. This can be seen to relate indirectly to resource availability and funding for making public areas and services more suitable for patients with disabilities and suggests a potential need for improvement.

Our patient characteristics that were identified were not comprehensive. For example, we did not have any data on obesity for our study, but some other studies have shown a relationship between obesity, SB, and the HRQOL, with a negative impact on various domains. Abresch et al. documented in his study that obesity had a significant impact in the QOL with lower scores found in the physical, social, and emotional domains [5]. Furthermore, there are no studies or evidence as to whether or not interventions to improve weight or fitness in children with SB would lead to a better HRQOL in the long run. In addition, this study did not have a healthy control group of children to compare the QOL. Therefore, recommendations for future studies would include these as part of their assessment.

The main drawback of this study is its relatively small sample size, which could have negatively and positively affected the power of the study, and therefore, some potentially significant differences may not have been identified. Possible reasons for the lack of patient participation could be the lack of interest by the parents or the lack of time or travel expenses. To reduce the percentage of dropouts due to financial reasons such as travel expenses, the option of home visitations was offered to parents via an invitation letter.

## Conclusions

Positive results were obtained regarding the medical, emotional, intellectual, and social aspects of patients with SB in Lithuania as they scored high in this domain. However, the environmental and vocational domains scored low, suggesting that further examination needs to be carried in these domains.

We concluded that having various comorbidities including hydrocephalus and incontinence has negative impacts on the QOL. Patients who suffered from epilepsy had a statistically significant lower QOL. No significant difference was found in the association between the level of lesion and the QOL in our study.

## Appendices

Health variables and the level of comorbidities questionnaire

1. Date of birth: ___________

2. Date of consultation: ________

3. Gender: 1. Male  2. Female

4. Lives with: 1. Father/one of the parents  2. Guardian  3. Care facility

5. Spinal cord injury level: 1. Chest  2. Waist L1-L2  3. Lumbar L3-L5  4. Sacral

6. Hydrocephalus (treated surgically): 0. No  1. How many times (if yes)

7. Chiari II malformations (treated surgically): 0. No  1. Yes

8. Syringomyelia (treated surgically): 0. No  1. Yes

9. Tethered spinal cord injury (treated surgically): 0. No  1. Yes

10. Epilepsy (treated with drugs): 0. No  1. Yes

11. Bone fractures: 0. No  1. How many times (if yes)

12. Bladder catheterization: 0. None  1. For a number of years (if yes)

13. Inserting bladder catheterization: 1. Alone  2. With help

14. Urinary incontinence: 0. None 1. Uses security measures sometimes  2. Always use protective equipment

15. Chronic pyelonephritis: 0. None  1. For a number of years (if yes)

16. Renal failure: 0. None  1. For a number of years (if yes)

17. Urology operation: 0. None  1. Bladder augmentation  2. Suprapubic cystoscopy  3. Bladder augmentation+suprapubic cystoscopy

18. Constipation: 0. None  1. Treated medically  2. Enema not regularly  3. Enema regularly (anterograde)  4. Enema regularly (retrograde)  5. Manual eradication

19. Walking (Hoffer scale): 0. Do not walks  1. Walks only during physiotherapy  2. Walks at home with aid  3. Walks in all environments with Aid  4. Walks normally

20. Spinal deformities: 0. None  1. Treated conservatively  2. Treated surgically

21. Hip deformities: 0. None  1. Treated conservatively  2. Treated surgically

22. Foot Deformities: 0. None  1. Treated conservatively  2. Treated surgically

23. Mental retardation: 0. None  1. Mild  2. Moderate  3. Severe  4. Very severe  5. Nonspecific
